# The Effectiveness of Psychological Therapies for Anxiety Disorders in Adolescents: A Meta-Analysis

**DOI:** 10.1007/s10567-021-00364-2

**Published:** 2021-09-01

**Authors:** Holly J. Baker, Peter J. Lawrence, Jessica Karalus, Cathy Creswell, Polly Waite

**Affiliations:** 1 School of Psychology and Clinical Language Sciences, University of Reading, Reading, RG6 6AL UK; 2 Radcliffe Observatory, University of Oxford, Anna Watts Building, Woodstock Rd, Oxford, OX2 6GG UK; 3 Centre for Innovation in Mental Health, School of Psychology, University of Southampton, Southampton, SO17 1BJ UK; 4 Central North West London NHS Foundation Trust, Grenfell Health and Wellbeing Service, St Charles Hospital, Exmoor Street, London, W10 6DZ UK

**Keywords:** Adolescent, Anxiety, Psychological treatment, Meta-analysis

## Abstract

Anxiety disorders are common in adolescence but outcomes for adolescents are unclear and we do not know what factors moderate treatment outcome for this age group. We conducted meta-analyses to establish the effectiveness of psychological therapies for adolescent anxiety disorders in (i) reducing anxiety disorder symptoms, and (ii) remission from the primary anxiety disorder, compared with controls, and examine potential moderators of treatment effects. The protocol was registered with PROSPERO (CRD42018091744). Electronic databases (Web of Science, MEDLINE, Psycinfo, EMBASE) were searched from January 1990 to December 2019. 2511 articles were reviewed, those meeting strict criteria were included. Random effects meta-analyses were conducted. Analyses of symptom severity outcomes comprised sixteen studies (CBT k = 15, non-CBT k = 1; *n* = 766 adolescents), and analyses of diagnostic remission outcomes comprised nine (CBT k = 9; *n* = 563 adolescents). Post-treatment, those receiving treatment were significantly more likely to experience reduced symptom severity (SMD = 0.454, 95% CI 0.22–0.69) and remission from the primary anxiety disorder than controls (RR = 7.94, 95% CI 3.19–12.7) (36% treatment vs. 9% controls in remission). None of the moderators analysed were statistically significant. Psychological therapies targeting anxiety disorders in adolescents are more effective than controls. However, with only just over a third in remission post-treatment, there is a clear need to develop more effective treatments for adolescents, evaluated through high-quality randomised controlled trials incorporating active controls and follow-up data.

## Introduction

Anxiety disorders are common in adolescence (Costello et al., 2011; Polanczyk et al., 2015; Vizard et al., 2018), with around 8% of 11- to 16-year olds (Vizard et al., 2018) meeting diagnostic criteria for an anxiety disorder. Anxiety disorders during this developmental stage are particularly problematic due to their negative impact on psychosocial functioning including education, social interaction, school refusal and school dropout (Van Ameringen et al., 2003). They are likely to persist into adulthood and are associated with a risk of developing other mental health problems (Pine et al., 1998), such as depression, alcohol dependence and suicidal behaviour (Kendall & Peterman, 2015; Kendall et al., 2004; Woodward & Fergusson, 2001), as well as negative long-term impairments in social and occupational functioning (Woodward & Fergusson, 2001). Consequently, the high prevalence and substantial impact of anxiety disorders during adolescence highlight the vital need for effective treatments.

There is a continuing growth in psychological treatments for child and adolescent anxiety disorders, including both cognitive behaviour therapy (CBT) and non-CBT-based approaches (e.g. mindfulness and acceptance-based therapies Dunning et al., 2019; Vøllestad et al., 2012), delivered in a range of formats such as individual, group and computer based (cCBT), in clinic and school settings, with varying degrees of parent/carer involvement. CBT is the most extensively evaluated treatment for anxiety disorders among children and adolescents, with generally good outcomes, across different formats of delivery. When outcomes have been examined across broad age ranges (2–19 years of age), 49.4% of children and adolescents who have had CBT (not including cCBT) have been found to be in remission from their primary anxiety disorder at the end of treatment (James et al., 2020).

It is unclear, however, to what extent these findings can be generalised to adolescents with anxiety disorders, who have typically been underrepresented in treatment outcome studies. Reynolds et al. (2012) examined the six studies in their review that recruited only adolescents (aged 14–19 years) with either elevated anxiety symptoms or an anxiety disorder diagnosis and found the reduction in symptoms post-treatment to be in the very large range (*d* =  − 1.38), although with very wide confidence intervals (95% CI 2.65, − 0.11). Although specific outcomes for the remission of anxiety disorders in adolescents are not reported, in two meta-analyses (Bennett et al., 2013; James et al., 2020) that have examined age as a moderator of outcome, they found no significant differences between studies comparing remission of anxiety disorders for adolescents and younger age groups. While James et al. (2020) found larger treatment effects for CBT (vs. no waitlist/ no treatment) among adolescents aged 12 years or more compared to children 12 years or less, they highlighted the substantial heterogeneity in findings. The majority of the studies in their review used mixed child and adolescent samples, with less than 20% of included studies focussing specifically on adolescents.

Examining adolescents in their own right is important, as adolescence is a unique stage of development and factors associated with this developmental period may influence the effectiveness of treatment for anxiety disorders. Findings from both animal and human research suggest that during adolescence, fear expression and extinction are temporarily impaired (Ganella & Kim, 2014; Waters et al., 2017) making it more difficult to retain new, non-fearful, inhibitory information. In addition, adolescents may have severe symptoms, comorbid depression and difficulty attending school (Hudson et al., 2002; Waite & Creswell, 2014). Taken together, these factors may influence the overall effectiveness of treatment and questions about what works for whom. Notably, to date, no studies have gone beyond examining age as a moderator to investigate *what* moderates outcomes for adolescents.

Factors that have been found to be associated with better treatment outcomes across broad age ranges include having a greater number of treatment sessions (i.e. more than 11 sessions), (Reynolds et al., 2012), treatments targeting a specific disorder rather than being transdiagnostic (Reynolds et al., 2012), clinical treatment-seeking populations rather than those recruited from the general community (Weisz et al., 2015), being from White ethnic backgrounds compared to those from other ethnic groups (Weisz et al., 2017) and comparisons with wait list controls, rather than active controls or treatment as usual (James et al., 2015, 2020; Reynolds et al., 2012). In contrast, poorer outcomes have been found for children and adolescents with social anxiety disorder than those with other anxiety disorders (Hudson et al., 2015). There have been mixed findings for delivery format; while Zhou et al., (2019) concluded that group formats (of CBT) are particularly effective compared to passive control groups as well as to most other psychotherapies, James et al. (2020) concluded that the evidence does not provide clear and consistent support for group CBT having an advantage over other delivery formats and highlighted that studies that differed in terms of treatment delivery format also differed on other key characteristics. Other factors, such as gender and parental involvement, have not significantly moderated treatment outcomes in studies to date (James et al., 2015; Manassis et al., 2014; Reynolds et al., 2012). Although children and adolescents from socio-economically disadvantaged backgrounds are significantly more likely to develop mental health problems than those from less disadvantaged backgrounds (Reiss, 2013; Reiss et al., 2019), whether this disadvantage specifically moderates treatment outcomes for anxiety disorders has not yet been examined. The extent to which these factors moderate outcomes specifically among adolescents has not been evaluated, however, there are clear developmental reasons that may lead to differences. For example, adolescent patterns of fear expression and extinction (Ganella & Kim, 2014; Waters et al., 2017) may lead to different effects based on the amount of treatment hours/sessions provided. Other factors such as the effectiveness of different modes of treatment delivery (e.g. individual, group or online) may also be unique in adolescence, due to their desire for autonomy (Zimmer-Gembeck & Collins, 2008), high levels of self-consciousness (Sebastian et al., 2008) and heightened sensitivity to others’ perceptions of themselves (Kilford et al., 2016). Understanding potential moderators of treatment within this unique developmental period is vital in understanding who does and does not benefit from psychological treatments for anxiety disorders to then develop more effective treatments in the future.

This meta-analysis aims to address the current gap in the literature by examining treatment outcomes and moderators of treatment outcome for adolescents with an anxiety disorder. It specifically focuses on RCTs of *any* psychological treatment (i.e. not just CBT-based approaches) using *any* delivery format (including cCBT), for anxiety disorders among adolescents. We defined the adolescent age range as 11–18 years (inclusive) based on 11 being the average age at which external indicators of puberty become apparent (American Psychological Association, 2002) and 18 being both the legal age of adulthood and the age at which child and adolescent mental health services end in many countries. In addition, typically 11–18 is the age range when young people are in secondary education, therefore adolescents in this age range have broadly similar educational and social demands and roles (Perry et al., 1993).

We aimed to answer the following research questions:

How effective are psychological therapies in (i) reducing anxiety disorder symptoms and (ii) achieving remission from the primary anxiety disorder, when compared with controls, at post-treatment and follow-up time points? (iii) Is the effectiveness of psychological therapies for treating anxiety disorders in adolescents moderated by the following treatment/demographic variables: CBT (including cCBT) vs non-CBT intervention, mode of treatment delivery (individual, group, mixed group and individual, cCBT), age, number of treatment hours, disorder-specific vs. generic anxiety treatment, active vs. passive control group, clinic vs. community sample, type of primary anxiety disorder, ethnicity (white or other ethnicity), gender (percentage female), parental involvement (involvement vs no involvement) and socio-economic status, at post-treatment and follow-up time points?

We also examined study quality as a moderator of treatment outcome. Very little is known about adverse events in RCTs of psychological treatments due to underreporting (Duggan et al., 2014). In evaluating the effectiveness of treatments, it is crucial to understand any potential harms as well as the benefits of therapy in terms of treatment outcomes. We therefore also examined the presence of adverse events reported within the identified studies. Finally, in addition to the aims specified in our protocol, we also examined to what extent interventions were developed or adapted to be developmentally sensitive to adolescents.

## Methods

**Fig. 1:**
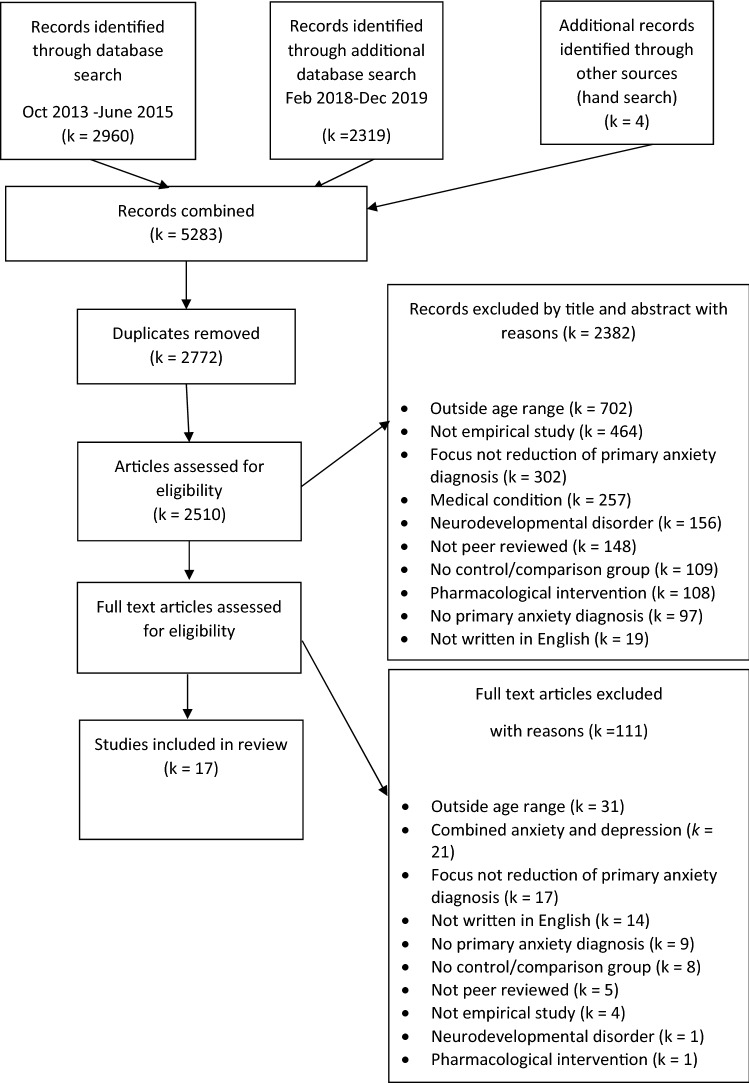
PRISMA diagram of study identification and selection

The review protocol was pre-specified and registered on the International Prospective Register of Systematic Reviews (PROSPERO; protocol number: CRD42018091744 https://www.crd.york.ac.uk/prospero/). PRISMA guidelines were followed throughout (Moher et al., 2009) (Fig. 1).

### Eligibility Criteria

To be included in the review each study had to meet the following criteria:

- Participants in the study were aged between 11 and 18 years of age (inclusive) at the start of treatment.
- Participants had a primary diagnosis of an anxiety disorder (with or without comorbid conditions).
- All diagnostic categories related to DSM-5 or ICD 10 anxiety disorders. Where studies involved participants with OCD or PTSD (no longer classified as anxiety disorders in DSM-5), they were only included where the percentage of participants with primary OCD or PTSD was each less than 10% of the total sample.
- Participants were randomly allocated to receive a minimum of one psychological treatment condition or one control condition.
- The study reported an outcome measure of anxiety symptoms and/or diagnostic status. Outcome measures were conducted at post-treatment or follow-up (any duration of follow-up was included).
- The study was published in peer-reviewed journals, in full text, from January 1990 onwards.
- The study was published in English. Non-English papers were documented but not included due to lack of resources for translation.

### Exclusion Criteria

Studies were excluded if they were:

- Studies of adolescents with medical conditions (e.g. diabetes, asthma).
- Studies of adolescents with learning disabilities or autism spectrum disorders.

These studies were excluded as psychological treatments for anxiety disorders would typically have been modified specifically for use with these populations, reducing comparability within the meta-analyses.

### Information Sources

The Web of Science and the NHS Healthcare databases incorporating results from MEDLINE, PsycINFO and EMBASE were used. Initial literature searches were conducted between October 2013 and June 2015. A supplementary search was run in February 2018, with a final search run in December 2019. Papers published between 1990 and December 2019 were included.

Search terms for psychological treatments were devised in line with those used by Reynolds et al. (2012), who examined both CBT and non-CBT treatments in their review. Key anxiety and child and adolescent terms were devised in line with James et al. (2015). We used the following anxiety key terms: anxiety, anxious, phobi*, “school refusal”, panic, mute, mutism, agoraphobi*. These terms were crossed with key terms relating to psychological treatment: treatment, therapy, psychotherapy, CBT, behaviour/ behaviour therapy, IPT and attachment and with key terms to identify studies using adolescents: child* or adolescen* or school* or p?ediatri* or young or youth*. Hand searching methods of reference lists of included papers were also carried out to identify additional studies of interest.

### Study Selection

Duplicate papers were removed after the initial database search. Two authors (HB and JK) independently screened titles and abstracts, comparing them against the inclusion/exclusion criteria. Papers were excluded on the basis of meeting none of the inclusion criteria or any of the exclusion criteria. The first criterion that was not met was recorded as the reason for rejection. Where grounds for exclusion were unclear, full texts were obtained and screened. Inter-assessor reliability for whether studies met inclusion criteria at the title and abstract stage was high (*Κ* = 0.82). Disagreements were discussed and reviewed by HB, JK, PW and CC to reach agreement. References were managed in Microsoft Excel and Endnote.

### Study Selection for Meta-Analysis

Inclusion in the meta-analysis at post-treatment or follow-up required studies to provide the number of participants in each condition, and either means and standard deviations or effect sizes for the intervention and control groups. Where standard errors were reported, these were converted to standard deviations. Where required data were unavailable, we contacted authors by email to request data. On completion of the selection process, 17 studies were eligible to be included in the review. See Fig. 1 for a PRISMA flowchart summarising the selection process (Moher et al., 2009).

### Data Extraction Process

Data extraction forms were developed prior to data extraction. The following data were extracted by author HB for all papers and by author JK for all papers published up to 2014: CBT versus non-CBT intervention, mode of treatment delivery (individual, group, mixed group and individual, cCBT), age, number of treatment hours, disorder-specific versus generic anxiety treatment, active versus passive control group, clinic vs. community sample, type of primary anxiety disorder, ethnicity (white or other ethnicity), gender (percentage female), parental involvement (involvement vs. no involvement) and socio-economic status. Information about adverse events in treatment was extracted by author HB.

For continuous outcome measures at all time points, adolescent self-report was preferred over parent report because adolescents are typically considered to be more accurate than their parents in reporting their anxiety symptoms (Cantwell et al., 1997). Where studies used multiple measures, the most commonly used self-report measures across studies were extracted. Where the trial intervention targeted mixed anxiety disorders, a generic, broad-based outcome measure of anxiety symptoms was extracted. For interventions targeting a specific anxiety disorder, disorder-specific outcome measures were extracted (if reported), consistent with previous meta-analyses (James et al., 2015, 2020). Where the most commonly used measure was not used in a study, the next most common measure was selected (see Table 1). Outcome data were independently extracted by a postgraduate psychology student and inter-assessor reliability was high (*Κ* = 0.85). The summary measure used for continuous symptom severity data was the standardised mean difference (SMD). SMD was defined as small (0.1), medium (0.3) or large (0.5) based on recommendations of Cohen (1992).

**Table 1:** Characteristics of individual studies included in the meta-analysis

Author, year	Age range (years)	Primary anxiety disorder	Diagnostic outcome measure	Sample size	Treatment	Sample recruited from	Type of control group	Outcome measure used for analysis	Parental Involvement	Treatment hours	% Female	Ethnicity % caucasian	Included participants on medication
Baer and Garland (2005)	13–18	SAD	–	11	Group CBT	Clinic	Passive	SPAI	Yes	18	58.3	–	Yes
Ebrahiminejad et al. (2016)	12–14	SAD	–	25	GMBCT	Community	Passive	SPIN	No	12	100	0%	No
Ginsburg and Drake (2002)	14–17	Mixed^a^	–	9	Group CBT	Community	Passive	SCARED	No	7.5	83.3	0%	–
Hayward et al. (2000)	14–17	SAD	ADIS-C/P	33	Group CBT	Community	Passive	SPAI	No	24	100	–	No
Herbert et al. (2009)	12–17	SAD	ADIS-C	68	Group CBT	Community	Active	SPAI-C	No	24^c^, 12^d^	56.0	47%	Yes
Ingul et al. (2014)	13–16	SAD	–	57	Group CBT Individual CBT, Mixed^b^	Community	Active	SPAI-C	No	10	56.1	–	–
Masia-Warner et al. (2005)	13–17	SAD	ADIS-C/P	35	Mixed	Community	Passive	SPAI-C	Yes	15.7	74.3	82.9%	No
Masia-Warner et al. (2007)	14–16	SAD	ADIS-C/P	32	Mixed	Community	Passive	SPAI-C	Yes	15.7	83.3	–	No
Masia-Warner et al. (2016)	14–17	SAD	ADIS-C/P	77	Group CBT	Community	Active	SPAI-C	Yes	18.5	68	72%	Yes
Olivares et al (2002)	15–17	SAD	-	59	Group CBT, individual CBT	Community	Passive	SAS-A	No	24^e^, 18^f^, 29	77.9	–	–
Pincus et al. (2010)	14–17	Panic disorder	-	26	Individual CBT	Clinic	Passive	MASC	Yes	9.2	19	100%	Yes
Spence et al. (2011)	12–18	Mixed	ADIS-C/P	115	Individual CBT, cCBT	Community	Passive	SCAS-C	Yes	10	59.1	–	–
Stjerneklar et al. (2019)	13–17	Mixed	–	67	cCBT	Community	Passive	SCAS-C	Yes	4	79	–	Yes
Swain et al. (2015)	12–17	Mixed	–	49	Group CBT, ACT	Clinic	Passive	MASC	No	15	63.3	67.3%	Yes
Waite et al (2019)	13–18	Mixed	ADIS-C/P	60	cCBT with therapist support	Clinic	Passive	SCAS-C	Yes	10	64.5	93.3%	Yes
Wuthrich et al. (2012)	14–17	Mixed	ADIS-C/P	43	cCBT	Community	Passive	SCAS-C	Yes	–^g^	62.8	77.3%	Yes

ADIS-C/P anxiety disorders interview schedule child and parent version, *ADIS-C* anxiety disorders interview schedule child version, *SAD* social anxiety disorder, *SPAI/SPAI-C* social phobia and anxiety inventory (child version), *SAS-A* social anxiety scale for adolescents, *SCAS-C* spence children’s anxiety scale, *MASC* multidimensional anxiety scale for children, *CGAS* children's global assessment scale, *SPIN* social phobia inventory, *SCARED* screen for child anxiety-related disorders, *GMBCT* group mindfulness-based cognitive therapy, *ACT* acceptance and commitment therapy, *CBT* cognitive behavioural therapy, *cCBT* computer-based CBT

^a^Mixed anxiety disorders

^b^Mix of individual and group sessions delivered

^c^Group

^d^Individual

^e^CBGT-A

^f^Social effectiveness therapy for adolescents (SET-A)

^g^Not reported

Diagnostic outcome data for primary anxiety disorder were extracted for all participants regardless of whether they completed treatment or not [i.e. treatment completers and intention to treat (ITT)], dependent on available data. All studies used the anxiety disorders interview schedule (ADIS-C/P; Albano & Silverman, 1996) to assess adolescent’s diagnostic status post-treatment. We chose to extract data on the number of patients free of their primary anxiety disorder rather than the number free of *all* anxiety disorders for both the intervention and control groups in order to examine recovery from the most impairing disorder, and because this is most commonly reported as the primary outcome in studies and other reviews and meta-analyses (James et al., 2015). The summary measure used was the risk ratio (RR) (Higgins & Green, 2011). RR were defined as small (1.22), medium (1.86) or large (3.00) based on guidelines set out by Olivier et al. (2017).

### Risk of Bias (Study Quality) in Individual Studies

Risk of bias was rated by author HB and independently second rated by author JK, using the Cochrane collaboration risk of bias guidelines for assessing studies (Higgins et al., 2011). Each assessment domain was scored as high, low or unclear. Where there was disagreement, this was discussed between raters, and a joint consensus was reached.

### Data Analysis

The R statistical environment was used for analysis. We used the ‘robumeta’ package for primary and moderator analyses (Fisher & Tipton, 2015). Where trials had more than one intervention group in a study (e.g. two different psychological interventions, compared to the same control condition), all interventions were included in the analysis. Because this violates the assumption of independence of data in meta-analysis, we used robust variance estimation, which corrects studies’ standard errors to account for associations between effects within studies (Hedges et al., 2010), so that we could examine all reported effects. We conducted random effects meta-analyses separately for studies that measured outcomes for (i) anxiety symptoms (continuous data) and (ii) remission of primary anxiety disorder (dichotomous data). For diagnostic outcomes, we conducted separate analyses of ITT data and treatment completer data. Where a study did not report ITT data (*k* = 8), this was calculated conservatively by assuming that all participants who dropped out of the index treatment still met diagnostic criteria and participants who dropped out of the control group were assumed to no longer meet diagnostic criteria. However, it was not possible to calculate missing data in this way for studies reporting symptom severity outcomes where ITT was not reported (data were unavailable in half the studies *k* = 8). We were therefore unable to conduct separate analyses of ITT and treatment completer data for symptom severity outcomes.

Planned moderator analyses (meta-regression) were completed only where there were more than ten studies in the meta-analysis (Higgins & Green, 2011). As there were only nine studies in the meta-analysis of diagnostic remission data, meta-regression was only carried out for symptom severity outcomes.

The ‘robumeta’ package used in meta-regression for dependent effect sizes applied the Satterthwaite (Satterthwaite, 1946) approximation to adjust for small samples (k). However, the assumptions of this approximation are not met when the degrees of freedom are < 4, therefore results run with degrees of freedom < 4 are unreliable (Fisher & Tipton, 2015; Tipton, 2015). Where results were identified as unreliable for this reason, they are identified in Table 2. In line with other similar meta-analyses (James et al., 2015; Warwick, 2017), all studies, regardless of their risk of bias status, were included in the analyses. Publication bias was assessed using funnel plots with the Egger statistical test of asymmetry for continuous and dichotomous data (Egger et al., 1997). However, in line with Cochrane guidance (Higgins & Green, 2011) that funnel plots should only be run if there are more than 10 studies in the analysis, this was only done for the analysis of symptom severity outcomes. To examine the impact of individual studies and publication bias on results, sensitivity analysis was conducted using the Vevea and Woods weight-function model (Vevea & Woods, 2005) and the ‘weightr’ package in R. The impact of statistical heterogeneity was measured using the *I*^2^ statistic (Higgins et al., 2003).

**Table 2:** Moderator analysis data for symptom severity outcomes

Moderating variable	Subgroup analysis			Moderator test	
	ES (*d*)	95% CI	*df*	Test statistic	*p* value
Intervention type (CBT vs. non-CBT)	0.075	− 0.171, 0.321	13.6	QM_1_ = 2.121	0.145
Treatment delivery				*F*_4.73_ = 3.73	0.100
Group	0.482	0.113, 0.852	5.81		
Individual^a^	− 0.575	− 1.258, 0.109	4.94		
Mixed^a^	0.557	− 0.455, 1.569	3.30^b^		
cCBT	− 0.130	− 0.624, 0.365	6.79		
Age				*t*_5.21_ = 0.340	0.747
Treatment hours				*t*_5.43_ = 1.300	0.246
Treatment type (specific vs. generic)	0.18	− 0.304, 0.669	12.28	QM_1_ = 1.243	0.265
Control group (active vs. passive)	0.356	− 0.112, 0.823	3.20^b^	QM_1_ = 2.121	0.145
Sample (community vs. clinic)	− 0.015	− 0.648, 0.619	4.37	QM_1_ = 0.035	0.851
Primary AD type (mixed vs. specific)	− 0.19	− 0.688, 0.314	10.27	QM_1_ = 1.234	0.267
Ethnicity (% Caucasian)				*t*_*2.79*_ = − 0.0444	0.968
Gender (% female)				*t*_3.83_ = 0.146	0.892
Parental involvement (involved vs. not)	0.027	− 0.506, 0.559	11.70	QM_1_ = 0.582	0.445
Study quality				*F*_3.25_ = 2.63	0.209
Poor^a^	0.713	0.343, 1.083	7.39		
Fair^a^	− 0.520	− 1,008, − 0.032	9.75		
Good	− 0.393	− 1.354, 0.567	1.89^b^		

*CBT* cognitive behavioural therapy, *cCBT* computer delivered CBT, *CI* confidence interval, *d* Cohen’s *d*, *df* degrees of freedom, *ES* effect size

^a^Within each moderator having more than 2 subgroups, identical superscript a indicates significant (*p* < 0.05) pairwise comparisons between subgroups

^b^Where subgroup variables were run with *df* < 4, they did not meet statistical assumptions for small sample adjustments and are therefore unreliable

Less than a third of studies reported any data for the intervention and control groups at follow-up (*k* = 5), with four studies reporting symptom severity data (Hayward et al., 2000; Herbert et al., 2009; Masia Warner et al., 2016; Olivares et al., 2002), and three studies reporting diagnostic data (Herbert et al., 2009; Masia Warner et al., 2016; Spence et al., 2011) at follow-up. Where follow-up data were available, a range of time points were reported (e.g. 6 or 12 months post-treatment), which led to a very small number of studies in each follow-up time point subgroup. Because precision of estimates can be adversely affected by small numbers of studies within analyses (Borenstein et al., 2011), we were unable to conduct meaningful analyses of follow-up data (Higgins & Green, 2011). Additionally, just seven out of 16 studies reported participants’ socio-economic status, and due to measurement and reporting differences relating to income, we were unable to analyse this variable. Therefore, the final moderating variables examined were CBT vs non-CBT intervention, mode of treatment delivery (individual, group, mixed group and individual, cCBT), age, number of treatment hours, disorder-specific versus generic anxiety treatment, active versus passive control group, clinic versus community sample, type of primary anxiety disorder, ethnicity (white or other ethnicity), gender (percentage female), parental involvement (involvement vs no involvement) and socio-economic status and study quality (risk of bias; poor, fair or good).

## Results

Database searches yielded a total of 5283 records. After removing duplicates and screening, a final total of 17 studies were assessed as eligible for inclusion. One study (O'Brien et al., 2007) could not be included in the meta-analysis because insufficient data (including the number of participants and means and standard deviations for intervention and control conditions post-treatment) were reported in the paper and we were unable to obtain the necessary data from the authors. Therefore, 16 studies were included in the analysis of continuous symptom severity outcomes (Baer & Garland, 2005; Ebrahiminejad et al., 2016; Ginsburg & Drake, 2002; Hayward et al., 2000; Herbert et al., 2009; Ingul, 2014; Masia-Warner et al., 2007; Masia-Warner et al., 2005; Masia Warner et al., 2016; Olivares et al., 2002; Pincus et al., 2010; Spence et al., 2011; Stjerneklar et al., 2019; Swain, 2015; Waite et al., 2019; Wuthrich et al., 2012), with a total sample of 766 adolescents. Nine studies were included in the meta-analysis of dichotomous remission data (Hayward et al., 2000; Herbert et al., 2009; Masia Warner et al., 2016; Masia-Warner et al., 2005, 2007; Spence et al., 2011; Stjerneklar et al., 2019; Waite et al., 2019; Wuthrich et al., 2012) with a total sample of 563 adolescents. Eight studies could not be included as they did not report remission data for the primary anxiety disorder (Baer & Garland, 2005; Ebrahiminejad et al., 2016; Ginsburg & Drake, 2002; Ingul, 2014; O'Brien et al., 2007; Olivares et al., 2002; Pincus et al., 2010; Swain, 2015).

Further information about the characteristics of included studies is provided in Table 1. Over half the studies (*k* = 9) had samples consisting of adolescents with a primary diagnosis of SAD. Three quarters (*k* = 12) recruited participants from the community (e.g. schools or advertisements), the remaining four were clinic samples. Fifteen studies looked at CBT (including one study of mindfulness-based cognitive therapy), with only one non-CBT study of acceptance and commitment therapy (ACT). Nine delivered CBT in a group, four delivered individual sessions, two combined group and individual sessions, while four studies examined cCBT. The number of treatment hours delivered ranged from 4 to 29 h. Half the studies (*k* = 8) used and reported ITT analysis for diagnostic outcomes (Ingul, 2014; Masia-Warner et al., 2007; Pincus et al., 2010; Spence et al., 2011; Stjerneklar et al., 2019; Swain, 2015; Waite et al., 2019; Wuthrich et al., 2012).

### How Effective are Psychological Therapies in Reducing Anxiety Disorder Symptoms?

**Fig. 2:**
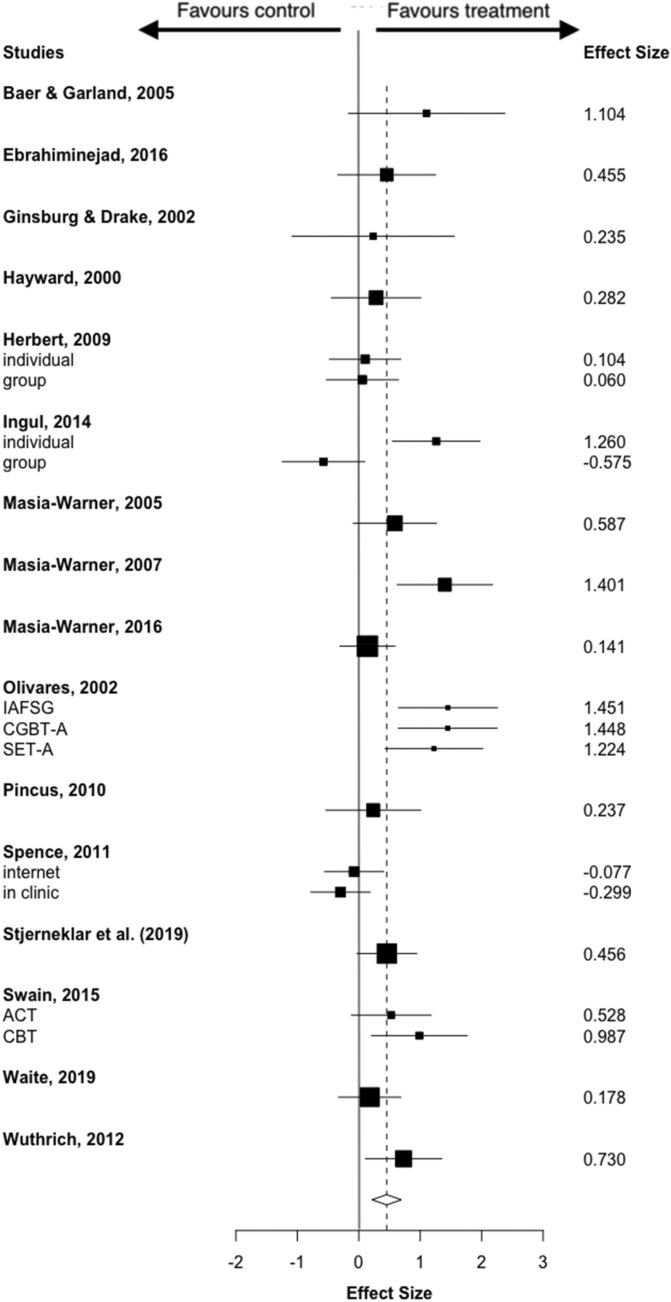
Forest plot of continuous outcomes

**Fig. 3:**
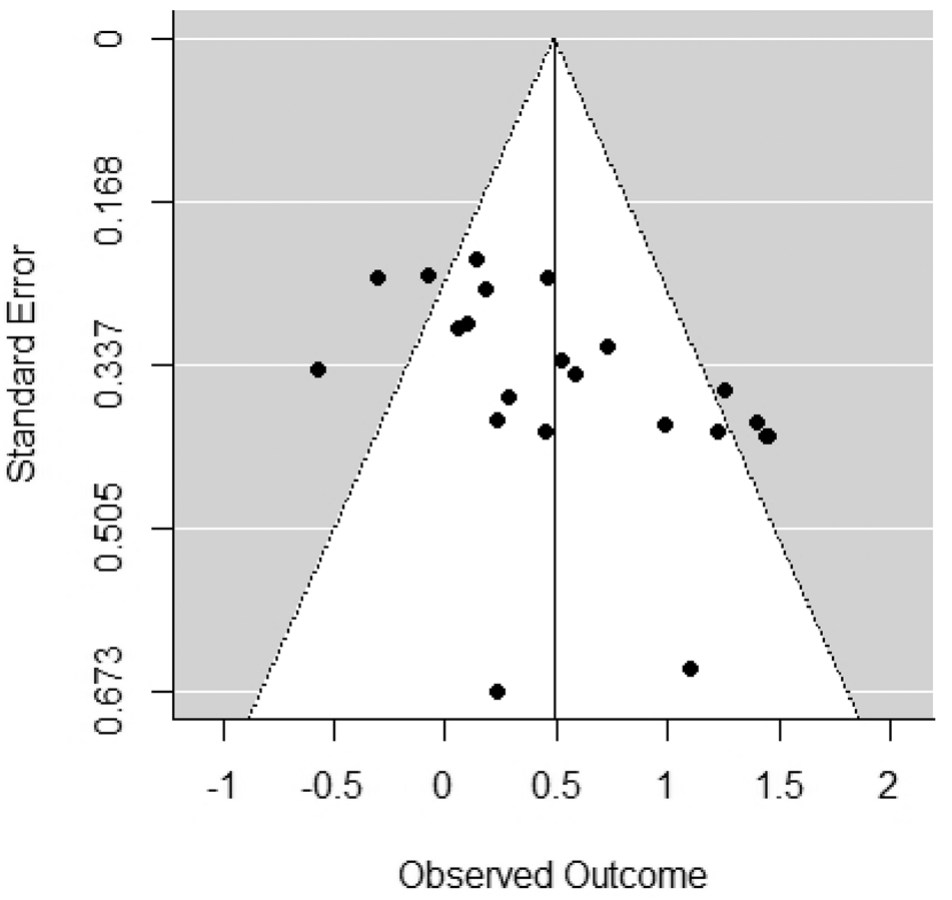
Funnel plot of continuous outcomes

Adolescents who had received psychological treatment reported a significantly greater reduction in symptom severity than controls, with a moderate effect size (SMD = 0.454, 95% CI 0.22–0.69), although there was moderate heterogeneity between studies (*I*^2^ = 53.56%) (Fig. 2). Visual inspection of the funnel plot revealed evidence of an asymmetrical distribution of studies (Fig. 3), and Egger test results were significant (*z* = 2.76, *p* = 0.051), consistent with publication bias. Sensitivity analysis using the Vevea and Woods’ weighted function model (Vevea & Woods, 2005) revealed that estimates for continuous outcomes (SMD = 0.37–0.51) proved robust and therefore it is unlikely that publication bias influenced results. High heterogeneity and poor study quality are potential sources of bias and as such may account for bias identified within funnel plot asymmetry in this analysis (Higgins & Green, 2011).

### How Effective are Psychological Therapies in Achieving Remission from the Primary Anxiety Disorder?

**Fig. 4:**
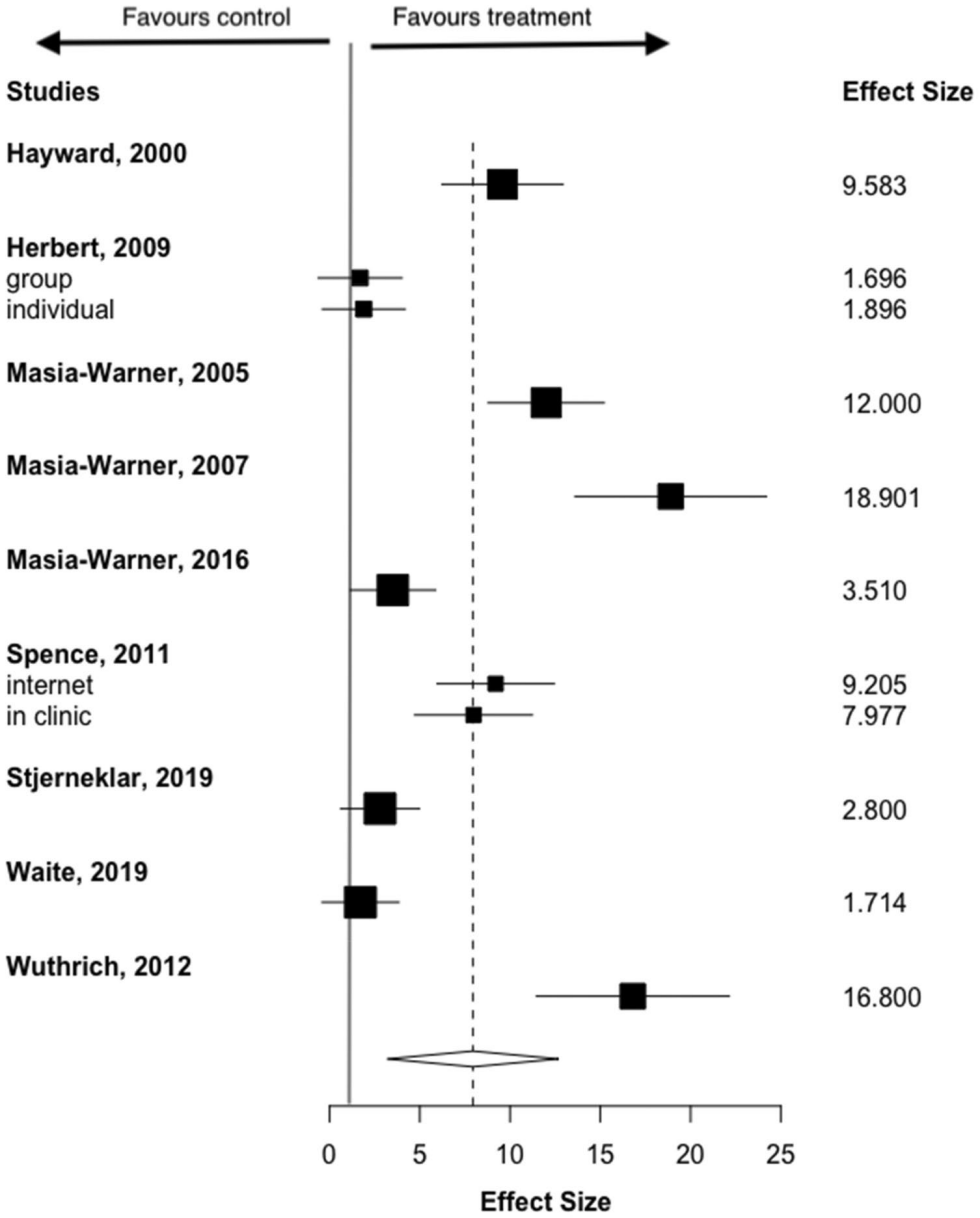
Forest plot of dichotomous outcomes: intention to treat (ITT)

**Fig. 5:**
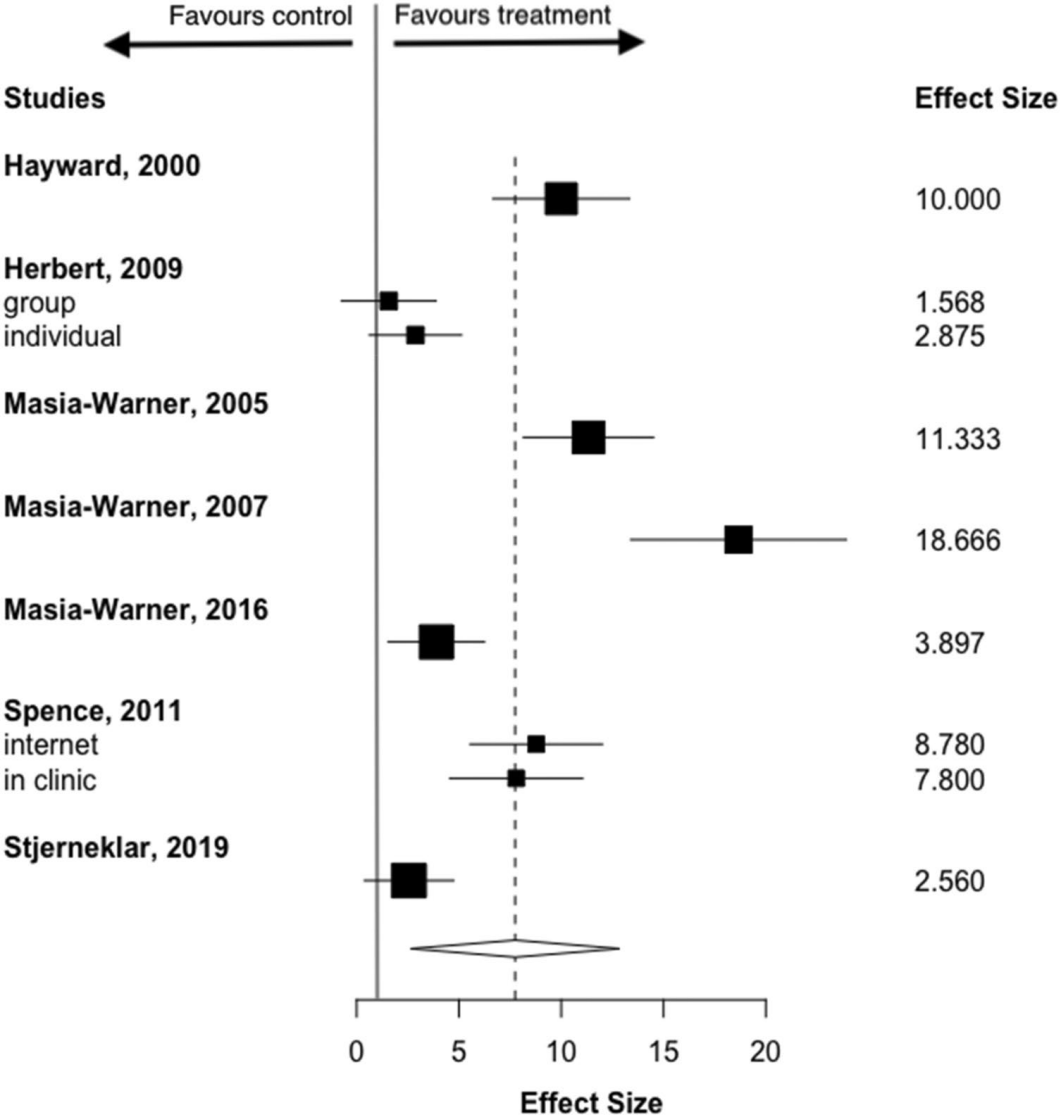
Forest plot of dichotomous outcomes: treatment completers

At post-treatment, remission from the primary anxiety disorder was significantly more likely among those in the psychological treatment group (ITT), compared to controls (RR = 7.94, 95% CI 3.19–12.7) equating to 36% (*n* = 116) of those in the treatment group (all CBT) versus 9% (*n* = 22) of controls being in remission post-treatment, although there was high heterogeneity (*I*^2^ = 91.7%) (Fig. 4). A similar pattern was found for treatment completers (RR = 7.21, 95% CI 3.83–10.58), equating to 37% (*n* = 94) of those in the CBT group versus 9% (*n* = 15) of controls in remission post-treatment, again, with high heterogeneity (*I*^2^ = 92.3%) (Fig. 5). We used Vevea and Woods’ weight-function model (Vevea & Woods, 2005) to analyse sensitivity. The estimates for ITT outcomes (RR = 6.21–8.10) and completer outcomes (RR = 5.93–7.93) proved robust, meaning that publication bias was an unlikely influence on results.

### Is the Effectiveness of Psychological Therapies for Treating Anxiety Disorders Affected by Moderating Factors?

Meta-regression was only carried out for symptom severity outcomes as there were fewer than 10 studies (*k* = 9) in the meta-analysis of diagnostic remission data.

A summary of the moderator analyses (meta-regression) findings can be found in Table 2. Meta-regression analyses found that none of the treatment/demographic moderators analysed were significantly associated with symptom severity outcomes: CBT versus non-CBT, mode of treatment delivery, age, treatment hours, disorder-specific versus generic anxiety treatment, active versus passive control group, clinic versus community sample, type of primary anxiety disorder, ethnicity, gender or parental involvement. Although treatment delivery was not a significant moderator, subgroup analyses revealed that mixed delivery was associated with significantly larger effects than individual delivery. However, this result may be unreliable, because assumptions of small sample size adjustments were not met for the ‘mixed’ subgroup (Tipton, 2015).

### Study Quality

**Fig. 6:**
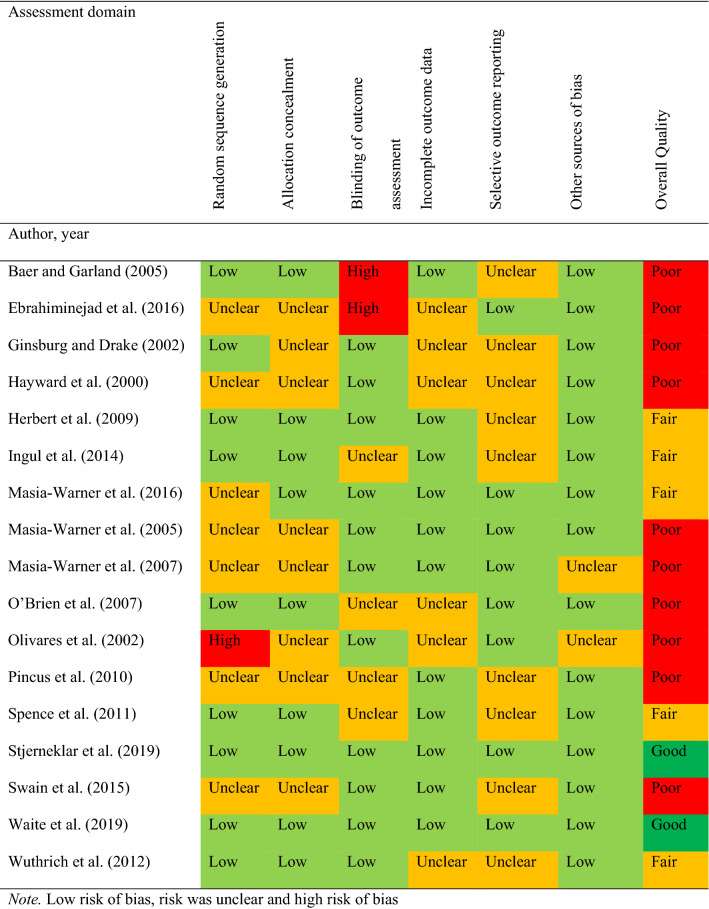
Cochrane Risk of bias assessment

Results of the risk of bias (study quality) assessment are presented in Fig. 6. Of the 16 included studies, only two (13%) were rated as ‘good’ quality overall, five (31%) were rated as ‘fair’ and nine (56%) rated as ‘poor’. Meta-regression analysis found study quality was not a significant moderator of symptom severity outcomes. Subgroup analyses revealed that ‘poor’ study quality was associated with larger effect sizes than ‘fair’ quality studies. We were unable to reliably analyse ‘good’ study quality as we could not meet the assumptions of small sample size adjustments (Tipton, 2015).

### Developmental Considerations

Although 13 of the 17 studies identified in the review reported using a developmentally adapted treatment, only six studies (37.5%) reported what aspects of the treatment were developed or adapted to be developmentally sensitive to adolescents (Ginsburg & Drake, 2002; Ingul et al., 2014; Masia-Warner et al., 2005; Pincus et al., 2010; Spence et al., 2011; Swain et al., 2015). Where modifications were described, this involved making language ‘age-appropriate’ (e.g. cognitive restructuring changed to ‘being a detective’ or ‘reality checking’), giving adolescent-specific examples (e.g. dating, at a party, or related to exams) or conducting exposure/social skills training within activities typical for adolescents (e.g. in a shopping centre or playing billiards). In three studies it was unclear whether a treatment designed for adolescents was used (Baer & Garland, 2005; Ebrahiminejad et al., 2016; Herbert et al., 2009).

### Adverse Event Reporting

Only two of the 16 studies (12.5%) reported adverse events that caused young people to drop out of the trial. In one study (Baer & Garland, 2005), a first episode of psychosis occurred during treatment, and in the second study (Waite et al., 2019), two participants in the waitlist condition had elevated risk of suicidality during the waitlist period.

## Discussion

Examining adolescents in their own right is important, as adolescence is a unique stage of development and factors associated with this developmental period may influence the effectiveness of treatment for anxiety disorders. We identified sixteen RCTs that examined the effectiveness of psychological treatments for anxiety disorders specifically in the adolescent age range. For adolescents who had completed a psychological treatment, compared to controls, there was a moderate and significant effect on symptom severity post-treatment. Just over half the studies examined remission from the primary anxiety disorder and both those randomised to, and those who completed, a psychological treatment were significantly more likely than controls to be in remission from their primary anxiety disorder post-treatment, with large effects. Despite this apparent positive finding, in the treatment group, only 36% of adolescents no longer met diagnostic criteria for their primary anxiety disorder at the end of treatment. We were unable to identify any treatment/demographic moderators that were significantly associated with outcomes.

There are a number of reasons to be cautious, however, when drawing conclusions from the available studies. Of concern, over half the studies were rated as ‘poor’ quality. Although study quality was not a significant moderator of symptom severity outcomes, our subgroup analyses revealed poor study quality was associated with larger treatment effects than those of fair quality, indicating that biases may have led to overestimated treatment effects. Forest plots showed high heterogeneity between studies and we were unable to statistically identify the source of this. Furthermore, there are limits to the extent that findings can be generalised given that three quarters of the studies recruited participants from the community (e.g. through schools) and half focussed specifically on social anxiety disorder. Conclusions cannot be drawn about the relative efficacy of different treatment types, as CBT (delivered in a variety of formats) was the treatment approach in all but one study.

Nevertheless, the reason why only a third of adolescents are free of their primary anxiety disorder at the end of treatment warrants urgent and extensive evaluation. This may reflect severe anxiety symptoms/disorders, high levels of social anxiety disorder/symptoms, comorbid depression, and potentially chronic and entrenched problems (Essau et al., 2000; Kendall & Peterman, 2015; Pine et al., 1998; Waite, 2014; Woodward & Fergusson, 2001), that do not respond sufficiently to current treatments. There is some evidence that adolescents have difficulty retaining new non-fearful information during this developmental stage (Waters et al., 2017). This could potentially account for adolescents’ poor responses to predominantly exposure-based anxiety treatments in the ways that they are currently delivered. Clearly, treatment optimisation must be underpinned by a clear developmental understanding of the mechanisms that maintain anxiety disorders in adolescents. However, the role of the wider context that adolescents are living in, including acute social demands and academic pressures (Blakemore, 2008, 2018), are also likely to be important. It was notable that less than half the studies reported what aspects of the treatment were designed to be developmentally sensitive to adolescents. Where adaptations were made, this consisted of making language ‘age-appropriate’, giving adolescent-specific examples, or conducting exposure/social skills training within activities typical for adolescents. It would be helpful for future studies to explicitly report how interventions have been developed or adapted to consider specific developmental needs.

Notably, none of the treatment and demographic variables previously shown to moderate the effectiveness of treatment when examined among children *and* adolescents, i.e. group delivery format (Zhou et al., 2019); greater number of treatment hours, disorder-specific treatment, type of control (Reynolds et al., 2012); and ethnicity (Weisz et al., 2017) moderated treatment effects specifically in adolescents. Given that adolescents often have severe symptoms, it was of interest that the number of treatment hours did not significantly moderate outcomes. However, studies that differed in treatment length differed on other key characteristics, making it difficult to draw meaningful conclusions. For example, all five studies where the treatment was ≥18 h were with adolescents with social anxiety disorder, which is typically associated with poorer outcomes (e.g. Hudson et al., 2015). Although disorder type was not a significant moderator, we were unable to examine associations with specific anxiety disorders, as all but one study focussed on social anxiety disorder or mixed anxiety disorders including social anxiety disorder. Mode of delivery was also not found to significantly moderate outcomes. Notably, two studies in this meta-analysis compared group and individual CBT directly, and found no significant differences in outcomes between delivery formats (Herbert et al., 2009; Ingul, 2014). However, both studies involved the treatment of adolescents with social anxiety disorder from the community (e.g. through schools) identified through screening and so it is possible that the young people in these studies were less severe than those referred to clinical services and potentially more responsive to working in a group format. Clearly, there is a great deal more work to be done to understand what works for whom, to then develop more effective treatments.

Unfortunately, we were unable to draw conclusions about potential adverse effects of treatment as only two studies reported adverse events. Clinical trials of psychological interventions have been identified as insufficiently reporting harm arising from treatment, as unlike with drug trials, this is not mandatory (Duggan et al., 2014). In a review of National Institute for Health Research (NIHR) funded trials, none of the psychological intervention studies reported adverse events in their final reports, and where adverse events were mentioned (e.g. within trial protocols), reporting guidelines for drug trials were used, which may not be suitable for psychological treatments (Duggan et al., 2014). To date, the focus of research examining psychological interventions has been on the benefits of therapy, but in future must also include the potential harm it might cause (e.g. worsening of symptoms, self-harm, suicide).

The strengths of this review include its specific focus on studies of the adolescent age range, examination of developmental adaptations used in treatments for adolescents, and examination of both diagnostic and symptom severity outcomes and potential moderators of symptom severity. For diagnostic outcomes, we were able to analyse ITT and completer data separately, allowing us to conclude that treatment effects were consistent across ITT and completer analyses for diagnostic outcomes. Nevertheless, our definition of the adolescent age range is a limitation that needs consideration. While we defined the adolescent age range as 11–18 years for the reasons outlined earlier, adolescence is an arbitrary definition and can be defined in multiple ways depending on the theoretical framework adopted (e.g. biological or psychosocial) (Curtis, 2015), anywhere between 9 and 26 years (American Psychological Association, 2002), with this upper end of the age span reflecting the neural development that continues beyond the age of 18 (Paus et al., 2008; Pfefferbaum et al., 1994). Older adolescents may have more in common with young adults than younger adolescents in terms of neurological development (Waters et al., 2017), and in the future, it will be important to consider the effectiveness of treatment for older adolescents and young adults, and at what stage adult-focussed treatment approaches become appropriate.

Results also need to be considered in light of several limitations of the included studies. The overall quality of studies in this review was poor. There were high levels of heterogeneity across study characteristics, outcome measures and reported outcomes (e.g. diagnostic remission status) and follow-up time points (where included). As pre-specified in our protocol, we only included studies that reported specifically on outcomes for adolescents aged 11–18 years, in order to examine the effectiveness of treatment and potential moderators of outcome during this unique stage of development. This also allowed us to examine to what extent interventions were developed or adapted to be developmentally sensitive to adolescents. Nevertheless, as a result of this approach, we are unable to draw direct conclusions about how the findings differ to those of children or adults, and therefore to what extent they are specific to adolescents. By selecting studies that only included adolescents, a large number of studies involving children and adolescents across broad age range were not included. Had we obtained data from these studies for participants within the 11–18-year age range, this is likely to have substantially increased the number of studies in the review and potentially made for a sample more representative of the wider literature, e.g. from a clinically referred population. Given the issues we have raised in this paper, where possible, we would encourage study authors to report outcomes separately for adolescent participants and provide open access to research data. Although we examined publication bias and found it unlikely to have had an impact on results, inclusion of only published works is a limitation, and we suggest future reviews include non-published works to address this. We analysed remission from primary anxiety diagnosis because this is the most commonly reported primary outcome measure in studies, however, it is likely the number of adolescents in remission from *all* anxiety diagnoses would be lower than the results of this meta-analysis show (Wuthrich et al., 2012) and Creswell et al. (2021) recommend that *all* anxiety disorders are assessed post-treatment and at follow-up in research trials. Very few studies were with clinically referred populations or active control groups. Furthermore, while 12 studies recruited participants from real world settings (e.g. schools), it remains unclear how generalisable the results of the studies are to adolescents with more severe clinical presentations of anxiety, who are seeking treatment in day or inpatient clinic settings. The majority of studies used passive or waitlist controls, potentially leading to an inflation of treatment effects, and there were insufficient studies to be able to conduct moderator analyses for diagnostic outcomes. We recommend that future studies fully report demographic factors (including socio-economic status and ethnicity) and participants’ clinical characteristics using consistent measures between studies to report baseline and treatment outcomes. In particular, we encourage the consistent use of assessment tools, outcome measures and reporting standards as set out by a recent international consensus statement on reporting treatment trials of child and adolescent anxiety disorders (Creswell et al., 2021). It is imperative that RCTs meet high methodological standards, and we recommend the use of active control groups, reporting of adverse events and reporting outcomes at follow-up to allow more rigorous examination of the effects of psychological interventions and potential moderating factors.

## Conclusion

Although there is evidence of efficacy of psychological therapies (predominantly CBT) targeting anxiety disorders in adolescents compared to (largely waitlist) controls, they have only a moderate effect on symptom severity and only just over a third of adolescents are in diagnostic remission after receiving treatment. Within the studies, we were unable to identify any moderators that influenced treatment outcome. There is a clear need to develop more effective treatments, that reflect adolescents’ specific developmental needs and that are evaluated through high-quality RCTs incorporating active controls and follow-up assessments to address the high prevalence, and substantial negative impact of adolescent anxiety disorders.
